# Indirubin modulates CD4^+^ T‐cell homeostasis via PD1/PTEN/AKT signalling pathway in immune thrombocytopenia

**DOI:** 10.1111/jcmm.14089

**Published:** 2019-01-04

**Authors:** Yajing Zhao, Panpan Han, Lei Liu, Xiaojie Wang, Pengcheng Xu, Haoyi Wang, Tianshu Yu, Yunqi Sun, Lizhen Li, Tao Sun, Xinguang Liu, Hai Zhou, Jihua Qiu, Liang Wang, Jun Peng, Shuqian Xu, Ming Hou

**Affiliations:** ^1^ Department of Haematology Qilu Hospital, Shandong University Jinan Shandong China; ^2^ Department of Urology Qilu Hospital, Shandong University Jinan Shandong China; ^3^ Department of Dermatology Qilu Hospital, Shandong University Jinan Shandong China; ^4^ Department of Geriatric Medicine Qilu Hospital, Shandong University Jinan Shandong China; ^5^ Shandong Provincial Key Laboratory of Immunohaematology Qilu Hospital, Shandong University Jinan Shandong China; ^6^ Key Laboratory of Cardiovascular Remodeling and Function Research Chinese Ministry of Education and Chinese Ministry of Health, Qilu Hospital, Shandong University Jinan Shandong China; ^7^ Leading Research Group of Scientific Innovation, Department of Science and Technology of Shandong Province Qilu Hospital, Shandong University Jinan Shandong China

**Keywords:** effector T cells, immune thrombocytopenia, programmed cell‐death 1, regulatory T cells

## Abstract

Immune thrombocytopenia (ITP) is an acquired autoimmune disease characterized by an immune mediated decrease in platelet number. Disturbance of CD4^+^ T‐cell homeostasis with simultaneous decrease of CD4^+^CD25^+^Foxp3^+ ^regulatory T cells (Tregs) as well as unrestricted proliferation and activation of peripheral CD4^+ ^effector T cells underpin the pathophysiology of ITP. Indirubin is an active ingredient of a traditional Chinese herb called *Indigofera tinctoria *L. which is clinically used for the treatment of ITP patients. Whether indirubin targets the Tregs/effector T cell‐axis to restore platelet number is unknown. In our in vitro studies, Indirubin could significantly enhance the number and function of Tregs and meanwhile dampen the activation of effector T cells in a dose‐dependent manner. Indirubin was observed to restore the expression of programmed cell‐death 1 (PD1) and phosphatase and tensin homolog (PTEN) on the CD4^+^ T cells of ITP patients, leading to the subsequent attenuation of the AKT/mTOR pathway. Furthermore, these observations were recapitulated in an active murine model of ITP with a prominent platelet response. Thus, our results identified a potentially novel mechanism of the therapeutic action of indirubin in the treatment of ITP through regulating the homeostasis of CD4^+^ T cells in a PD1/PTEN/AKT signalling pathway.

## 1 INTRODUCTION

Immune thrombocytopenia (ITP) is an autoimmune disease predominantly characterized by autoantibody‐mediated platelet destruction and/or impaired platelet production.^1^ Abnormalities in T‐cell populations are one of the major mechanisms for the pathogenesis of ITP.^2^, ^3^, ^4^ In ITP patients, platelet auto‐reactive CD4^+^ effector T cells are excessively activated with decreased apoptosis, while the CD4^+^ regulatory T cells (Tregs) are numerically and functionally impaired.^3^, ^4^, ^5^, ^6^, ^7^, ^8^ Skewed subsets of CD4^+^ T helper cells manifested as the imbalance of Th1/Th2, as well as elevated Th17, Th22 and Th9 levels were reported in ITP patients, contributing to a cytokine imbalance that can lead to lower levels and abnormal function of Tregs.^9^, ^10^, ^11^, ^12^, ^13^ Tregs play an important role in maintaining self‐tolerance and are phenotypically defined as CD4^+^CD25^+^Foxp3^+^. One facet of the pathophysiology of ITP and other autoimmune diseases involves deficiencies in number and function of peripheral Tregs.^14^, ^15^, ^16^, ^17^, ^18^, ^19^, ^20^, ^21^ It has been suggested that ITP murine models have retention of Tregs in the thymus, which may suppress the proliferation and function of Tregs in peripheral lymphoid organs, such as spleen and lymph nodes.^22^ Peripheral Tregs deficiencies could result in unrestricted proliferation and function of auto‐reactive effector T cells, contributing to the breakdown of immune homeostasis and autoimmmune diseases.^8^ The importance of T‐cell homeostasis in attenuating autoimmunity is further evidenced in the current clinical treatment of ITP. Both clinical and experimental studies show treatments such as intravenous immunoglobulin, dexamethasone, rituximab, and thrombopoietic mimetics, exert their therapeutic effects at least partially through modulating Tregs activation and expansion, and/or dampening the survival and proliferation of effector T cells.^14^, ^17^, ^22^, ^23^, ^24^

Indirubin is an active ingredient of a traditional Chinese herb called *Indigofera tinctoria *L. and is most well‐known for its clinical use in the treatment of chronic myelogenous leukemia.^25^ There are also reports of indirubin possessing anti‐viral, anti‐bacterial and anti‐inflammatory properties.^26^, ^27^, ^28^, ^29^, ^30^ In murine models of ITP and ulcerative colitis, indirubin was effective therapeutically through down‐regulating the immune response and increasing the percentage of Tregs.^27^, ^31^ Clinically, *I tinctoria* L. is used as a traditional Chinese medical therapy for thrombocytopenia patients. However, the efficacy of *I tinctoria *L. in the treatment of ITP still needs to be confirmed by appropriate clinical trials and the endogenous mechanisms of action remains to be explored.

Programmed cell‐death 1 (PD1) is a co‐receptor that is inducibly expressed on T cells. Tregs activation and expansion in parallel with PD1 signalling are critical for the maintenance of CD4^+ ^T‐cell homeostasis and elimination of either can result in its breakdown.^32^ Dysregulation of the PD1 pathway can be an important contributor to autoimmune pathogenesis, as has been shown in rheumatoid arthritis (RA), systemic lupus erythematosus, and multiple sclerosis (MS).^33^, ^34^, ^35^, ^36^, ^37^, ^38^ Furthermore, PD1 and its ligand PDL1 were reported to be decreased in the PBMCs of ITP patients, which suggested the important role of the PD1 pathway in the pathogenesis of ITP.^39^

Here we demonstrate that the therapeutic effects of indirubin may be achieved through the regulation of CD4^+^ T‐cell homeostasis in ITP by promoting the development and function of Tregs as well as reducing the activation of effector T cells via a PD1/phosphatase and tensin homolog (PTEN)/AKT signalling pathway.

## 2 MATERIALS AND METHODS

### 2.1 Patients and controls

A total of 36 consenting patients with ITP (19 females and 17 males; 15–77 years old; median age, 47) were enrolled between March 2016 and May 2018 in the Department of Haematology, Qilu Hospital, Shandong University, China. All ITP patients fulfilled the clinical diagnosis criteria recommended by the 2011 American Society of Haematology guidelines.^40^ The patient's platelet counts ranged between 1 × 10^9^/L and 95 × 10^9^/L, with a median count of 15 × 10^9^/L. The demographic and key clinical information of ITP patients are summarized in Table 1 and Table 2. Previous therapy were completed at least 3 weeks prior to enrollment. In parallel, 28 consenting healthy volunteers (13 females and 15 males; age range, 23–71 years; median age, 40 years) were recruited to establish healthy controls. And their platelet counts ranged between 143 × 10^9^/L and 358 × 10^9^/L, with a median count of 256 × 10^9^/L (Table 2). All study subjects donated 4 or 5 mL of heparinized venous peripheral blood. Our study was approved by the Institutional Review Boards of Qilu Hospital, Shandong University, China. All blood samples were collected after obtaining the informed consent from each participant before being included in the study.

**Table 1:** Clinical characteristics of ITP patients

Patient no.	Sex/age (y)	Platelet count (10^9^/L)	Course of disease (mo)	Antiplatelet antibodies		Major previous drugs	Bleeding symptoms
				Anti‐GPIIb/IIIa	Anti‐GPIb/IX		
1	F/61	20	5	−	−	DEX	PT, EC, GH
2	M/60	2	2	−	−	TPO	PT
3	M/45	33	3	−	−	None	None
4	F/26	17	3	−	−	None	None
5	F/36	2	5	+	−	DEX	EC
6	M/51	95	0	−	−	DEX	EC, GH
7	F/48	17	0	+	+	DEX	PT
8	F/46	13	2	−	+	Pred	GUH
9	M/21	8	12	+	−	DEX, TPO, Rit	None
10	M/39	26	84	−	−	DEX, TPO	EC, GH
11	M/60	8	72	−	−	TPO, Rit, IVIg	PT, GH
12	M/28	39	4	−	−	DEX	EC, GH, EP
13	F/61	65	260	−	+	DEX	EC, GH, EP
14	F/25	16	1.5	−	−	DEX	GH
15	M/75	9	1	+	+	None	GH
16	M/61	15	3	+	−	None	None
17	M/52	31	6	−	−	None	None
18	F/46	56	3	−	−	None	None
19	M/58	16	260	−	−	DEX, SP, TPO	PT, EC, GH
20	F/53	6	36	+	−	Pred	None
21	F/71	2	3	−	+	DEX	None
22	M/37	55	48	−	−	None	None
23	F/32	1	4	+	−	None	PT, EP
24	F/47	32	12	+	+	None	None
25	F/15	10	36	−	−	Pred, IVIg	PT, GH
26	M/64	16	2	+	−	DEX	EC, GH
27	M/58	3	120	−	−	TPO, IVIg	PT, EC
28	M/62	8	30	−	−	DEX, Rit, TPO	PT, GH, EP
29	F/19	8	12	−	+	DEX, TPO, Rit	PT, EC, GH
30	F/46	12	6	−	−	Pred, Olse	PT
31	F/27	2	108	−	+	DEX	EC
32	M/77	28	48	+	−	Pred, TPO	EP
33	F/52	23	1	−	−	Dex	EP
34	F/41	19	24	−	−	Dex	EC GUH
35	F/57	7	1	−	+	Dex, TPO	EC PT
36	M/24	12	0	−	+	TPO	NONE

EC, ecchymoses; EP, epistaxis; GH, gingival hemorrhage; GUH, genitourinary hemorrhage; ITP, immune thrombocytopenia; IVIg, intravenous gamma globulin; Olse, oseltamivir; Pred, prednisone; PT, petechiae; Rit, rituximab; SP, splenectomy; TPO, thrombopoietin.

The month number (n) in the course of disease was regarded as the disease course less than n + 1 mo.

**Table 2:** Baseline characteristics of ITP patients and healthy controls

	ITP patients	Healthy controls	*P* values
Median Age, y (range)	47 (15‐77)	40 (23‐71)	0.622
Gender, n (%)	17 (47.2%)	15 (53.5%)	0.614
Median platelet count, ×10^9^/L (range)	15 (1‐95)	256 (143‐358)	<0.0001

ITP, immune thrombocytopenia.

### 2.2 Mice and animal model

Wild‐type (WT) C57BL/6 mice were obtained from the Centre for New Drug Evaluation of Shandong University and served as platelet donors. Severe combined immunodeficient (SCID) mice with a C57BL/6 background (J001913, 6–8 weeks of age, male) were purchased from Jackson Lab (Bar Harbor, Maine, USA) and served as spleen‐cell transfer recipients. C57BL/6 CD61‐KO mice (B6.129S2‐Itgb3tm1Hyn/JSemJ; Stock No: 008819) were generously provided by Dr. Junling Liu from Shanghai Jiaotong University, School of Basic Medicine. For the establishment of ITP murine models, blood was drawn by retro‐orbital bleeding, and centrifuged at 60 *g* for 10 minutes to collect the platelet rich plasma (PRP). Platelets were gathered from PRP via centrifugation at 800 *g* for 5 minutes, and adjusted to a concentration of 10^9^/mL for immunization. C57BL/6 CD61 KO mice were transfused weekly with 100 µL of 10^8^ platelets from WT mice for three consecutive weeks. The immunized CD61 KO mice were euthanized, and their spleens were harvested and single‐cell suspension prepared.^1^ On the day of splenocytes transfer, SCID mice were subjected to 180 cGy total body irradiation to inhibit recipient rejection and enhance engraftment. Within 3 hours of irradiation, mice were injected intraperitoneally with 100 µL of the immunized splenocytes preparations (5 × 10^4^ splenocytes/mouse). (2ʹZ)‐indirubin (indirubin, Sigma‐Aldrich, St. Louis, MO, USA) was dissolved in corn oil (Sigma‐Aldrich) at 5 mg/mL and stored at 4°C. Immune thrombocytopenia murine models were randomly separated into two groups. The indirubin group mice received an intraperitoneal injection of indirubin (40 mg/kg) and the control group received the same volume of corn oil on the 13th day after irradiation (n = 6 and 7 for indirubin and control group respectively). Platelets were counted weekly using complete blood count after 1/10 dilution. The SCID mice were euthanatized 5 weeks after irradiation. Spleens, thymuses and thigh bones were removed to prepare single‐cell suspensions for flow cytometry. All animal experiments were performed under the approval of the Shandong University, Qilu hospital research ethics committee.

### 2.3 Isolation and culturing of PBMCs and CD4^+^ T cells

Peripheral blood mononuclear cells (PBMCs) from the patients and healthy controls were isolated from heparinized venous peripheral blood using Ficoll–Hypaque centrifugation (Amersham Biosciences, Piscataway, NJ, USA). Isolation of circulating CD4^+^ T cells was performed using anti‐CD4‐coated magnetic beads and MS column (Miltenyi Biotec, Bergisch Gladbach, Germany) separation. The purity of the isolated cells was found to be >90% by flow cytometry.

PBMCs or CD4^+^ T cells were resuspended in RPMI‐1640 medium (Life Technologies, Paisley, UK) supplemented with 10% heat‐inactivated fetal bovine serum (Gibco, Grand Island, NY, USA), 1% penicillin and streptomycin (Solarbio, Beijing, China), and recombinant human IL‐2 (5 ng/mL, R&D Systems, Minneapolis, MN, USA), anti‐human CD3 antibodies (1 ng/mL; eBioscience, San Diego, CA, USA), and anti‐human CD28 antibodies (1 ng/mL; eBioscience). The cells were then seeded on 24‐well plates.

Indirubin was dissolved in dimethyl sulfoxide (DMSO, Sigma‐Aldrich) to generate a stock solution of 5 mg/mL. Cultures of PBMCs or CD4^+^ T cells were treated with indirubin at a concentration of 0.01, 0.1, 0.2, 1, 2, and 10 µM, whereas 1‰ DMSO was used as vehicle controls. After 72 hours of incubation with indirubin or DMSO, PBMCs or CD4^+^ T cells were collected for flow cytometry, RNA extraction, and western blotting.

### 2.4 Flow cytometry

Tregs were detected using a regulatory T‐cell staining kit (eBioscience). Briefly, a total of 1 × 10^6^ cultured human PBMCs were harvested from 24‐well plates following respective treatments. For the preparation of mouse single‐cell suspensions, the spleens, thymuses and bone marrow from ITP mice were smashed and lysed using ACK lysing buffer to remove red blood cells. Cells were then washed two times by centrifugation at 300 *g* for 10 minutes and adjusted to a concentration of 1 × 10^6^ cells/mL for flow cytometry analysis. Subsequently, the single‐cell suspensions were stained as per manufacturer's instructions using anti‐human or anti‐mouse CD4, CD25, and Foxp3 conjugated‐antibodies (eBioscience).

The expression of PD1, PTEN and PDL1 was determined using anti‐human or anti‐mouse PD1 and PDL1 conjugated‐antibodies (eBioscience), and anti‐human/mouse PTEN (Miltenyi Biotec). Mouse antigen presenting cells (APCs) were identified with anti‐mouse CD80 and anti‐mouse CD86 conjugated‐antibodies (BD Biosciences, SanJose, CA, USA).

The activation of CD4^+^ T cells was measured by immunofluorescence staining using anti‐human CD4, CD25, CD45RA and CD45RO conjugated‐antibodies (eBioscience). For apoptosis analysis, PBMCs cultured with the respective doses of indirubin or 1‰ DMSO were washed twice with cold 1 × PBS and stained with FITC‐Annexin V and PI using a Cell Apoptosis Kit (Bestbio, Shanghai, China). Cells were counted using a FACS Calibur cytometer within 15 minutes after staining. Data analysis was carried out using FACS Calibur cytometer equipped with Kaluza Flow Cytometry Analysis Software (Beckman Coulter).

### 2.5 Suppression assay

Fresh CD4^+^CD25^−^ T cells and CD4^+^CD25^+^ Tregs were isolated from PBMCs using a CD4^+^CD25^+^ Regulatory T Cell Isolation Kit (Miltenyi Biotec) per manufacturer instructions. CD4^+^CD25^−^ T cells (effector T cells) were labelled with CFSE (5 µmol/L; Sigma‐Aldrich), seeded at 2 × 10^5^ cells/well, and then co‐cultured with or without Tregs at a ratio of 4:1 on a 96 well‐plate. The wells were also supplemented with IL‐2 (5 ng/mL, BD), anti‐human CD3 antibodies (1 ng/mL; eBioscience), and anti‐human CD28 antibodies (1 ng/mL; eBioscience). Sample data was acquired for flow cytometry analysis 6 days after cells incubation with 1 µmol/L indirubin or 1‰ DMSO. The data was analyzed using Flow Jo software.

### 2.6 Western blotting

Isolated CD4^+^ T cells cultured with 1 µmol/L indirubin or 1‰ DMSO were lysed in radio‐immunoprecipitation assay (RIPA) buffer (Bestbio). Immunoblots were performed using polyclonal rabbit anti‐human GAPDH (ZSGB‐BIO, Beijing, China), polyclonal rabbit anti‐human mammalian target of rapamycin (mTOR), p‐mTOR, AKT, p‐AKT, PTEN, and p‐PTEN antibodies (Cell Signaling Technology, NJ, USA). Semi‐quantitative evaluation of a particular protein's total level was analyzed by calculating the band of interest with respect to the housekeeping GAPDH signal. The level of phosphorylated proteins was measured using the ratio of phosphorylated protein to total protein. Results were expressed as fold increase relative to control (DMSO treated CD4^+^ T cells).

### 2.7 Quantitative real‐time PCR

Trizol reagent (Takara, Japan) was used to isolate total RNA from cultured CD4^+^ T cells. RNA was reverse transcribed into cDNA using the PrimeScript RT reagent kit (Perfect Real Time; Takara) according to the manufacturer's instructions. Multiplex real‐time RT‐PCR was performed for *PTEN* (forward: 5ʹ‐GCTGTGGTTGCCACAAAGTGCC‐3ʹ; reverse：5ʹ‐GCAGGTAGAAGGCAACTCTGCCA‐3ʹ), *PD1* (forward: 5ʹ‐CTCAGGGTGACAGAGAGAAG‐3ʹ; reverse：5ʹ‐GACACCAACCACCAGGGTTT‐3ʹ) and the endogenous control *GAPDH* (forward: 5ʹ‐GCACCGTCAAGGCTGAGAAC‐3ʹ; reverse: 5ʹ‐TGGTGAAGACGCCAGTGGA‐3ʹ) on a LightCycler^®^ 480 System (Roche Applied Science, Mannheim, Germany). In order to calculate relative changes in gene expression, target genes were compared to the housekeeping gene GAPDH using the comparative delta Ct ($\Delta \Delta C_{t}$) method.

### 2.8 Statistical analysis

Data were reported as mean values ±SD or Median with interquartile range, and were assessed for statistical significance using SPSS. Differences between pre‐ and post‐ treatment groups were determined by paired Student's *t* test and differences between two independent groups were compared using the Student's *t* test, and the data were expressed as mean values ±SD. For the data not normally distributed, the Wilcoxon matched‐pairs test and Mann‐Whitney test were used, and the data were expressed as Median with interquartile range. The *P* values <0.05 were considered to be of significance.

## 3 RESULTS

### 3.1 Indirubin increased the number and suppressive function of Tregs among ITP patients

**Figure 1:**
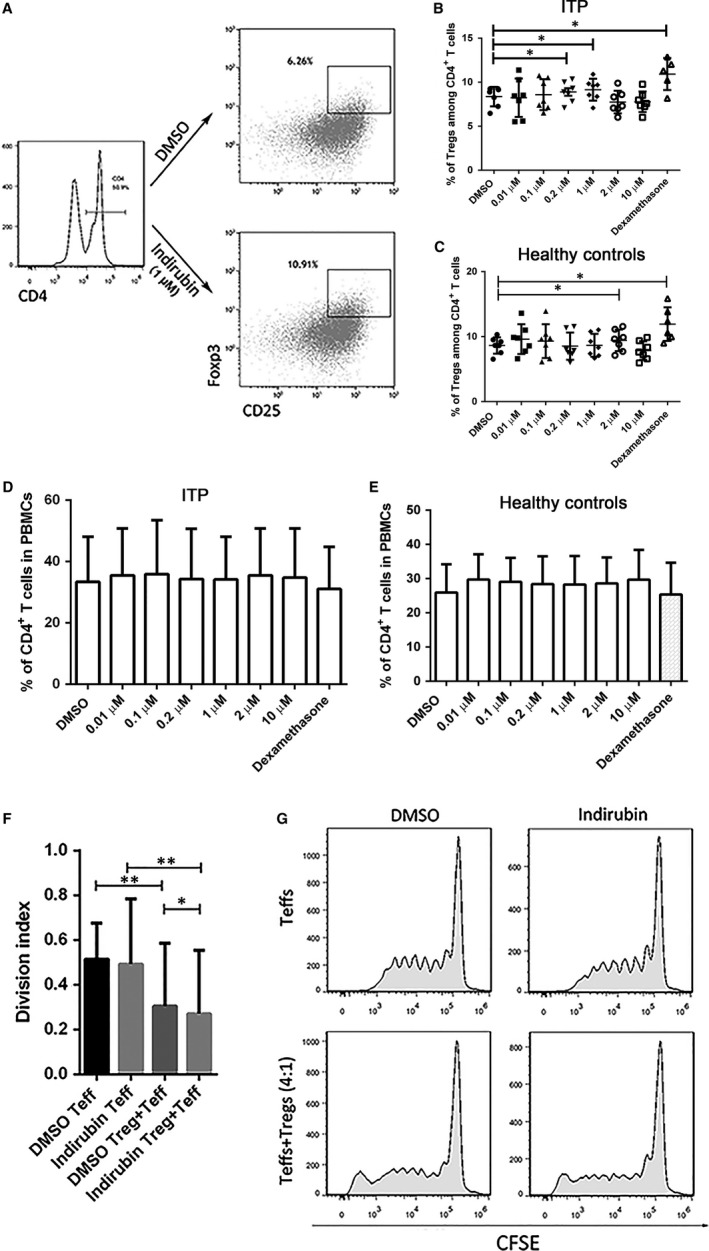
Indirubin increased the number and suppressive function of Tregs. (A) The gating strategy and representative dot plots for identification of CD4^+^CD25^+^Foxp3^+^ Tregs. (B) In the culturing of peripheral blood mononuclear cells (PBMCs) from immune thrombocytopenia (ITP) patients, the percentages of Tregs among CD4^+^ T cells were significantly increased after indirubin treatment at the dose of 0.2 and 1 µmol/L (8.37 ± 1.1 vs 8.89 ± 1.15, n = 7, *P* *=* 0.0335; 8.37 ± 1.1 vs 9.15 ± 1.25, n = 7, *P* = 0.0224). (C) In the culturing of PBMCs from healthy controls, the percentages of Tregs among CD4^+^ T cells were significantly increased after 2 µmol/L indirubin treatment (8.63 ± 1.26 vs 9.55 ± 1.87, n = 7, *P* = 0.0488). Dexamethasone was used as a positive control for the induction of Tregs (8.37 ± 1.1 vs 10.92 ± 1.82, n = 7, *P* = 0.0302 for ITP group; 8.63 ± 1.26 vs 11.93 ± 2.59, n = 7, *P* = 0.0333 for healthy control group). (D,E) The percentage of CD4^+^ T cells among the PBMCs after cultured with different doses of indirubin were not changed apparently in the ITP and healthy control groups. Significance between the two groups was determined by paired Student's *t* test. (F) The division index of effector T cells proliferation. Tregs significantly suppressed the proliferation of effector T cells both in the dimethyl sulfoxide (DMSO) and indirubin‐treated groups (DMSO Teff 0.52 ± 0.31 vs DMSO Teff+Tregs 0.31 ± 0.33, *P* = 0.0039; indirubin Teff 0.49 ± 0.41 vs indirubin Teff+Tregs 0.27 ± 0.32, *P* = 0.0039). Indirubin significantly enhanced the inhibitory function of Tregs compared with DMSO (*P* = 0.0117). (G) Representative histograms of CFSE dilution of CD4^+^CFSE^+^ effector T cells. Differences between two groups were determined by Wilcoxon matched‐pairs signed rank test, n = 9, *P < 0.05, **P < 0.01

Tregs were reported to be quantitively and functionally impaired in ITP patients, which was thought to contribute to disregulation of immune homeostasis.^3^, ^4^ It has been reported that indirubin could increase the number of Tregs as well as ameliorate thrombocytopenia in ITP murine models.^31^ Using PBMCs isolated from ITP patients and healthy controls, we tested whether indirubin could exert an influence on Tregs. In our study, indirubin could increase the number of Tregs in the culture of PBMCs from ITP patients at the dose of 0.2 and 1 µmol/L (Figure 1A,B), and PBMCs from healthy controls were most responsive to indirubin in the upregulation of Tregs at the dose of 2 µmol/L (Figure 1C). The percentages of CD4^+^ T cells among the PBMCs cultured were unchanged after respective treatments compared with DMSO controls (Figure 1D,E). Dexamethasone was used as a positive control for it was reported to enhance Tregs in the treatment of ITP.^24^ To explore whether indirubin could enhance the suppressive function of Tregs aside from increasing the number of Tregs, we co‐cultured CFSE‐labelled effector T cells with CD4^+^CD25^+^ Tregs isolated from ITP PBMCs in presence of either 1 µmol/L indirubin or 1‰ DMSO. For each sample run, the number of peaks was counted and the division index was calculated using Flow Jo software. Tregs markedly suppressed the proliferation of effector T cells in both groups. Furthermore, indirubin could significantly enhance the inhibitory function of Tregs compared with DMSO. Indirubin itself had no apparent effect on the proliferation of effector T cells, suggesting that indirubin could enhance the function of Tregs in suppression of the proliferation of effector T cells (Figure 1F,G).

### 3.2 Indirubin decreased fidelity of effector T cells in a dose‐dependent manner

**Figure 2:**
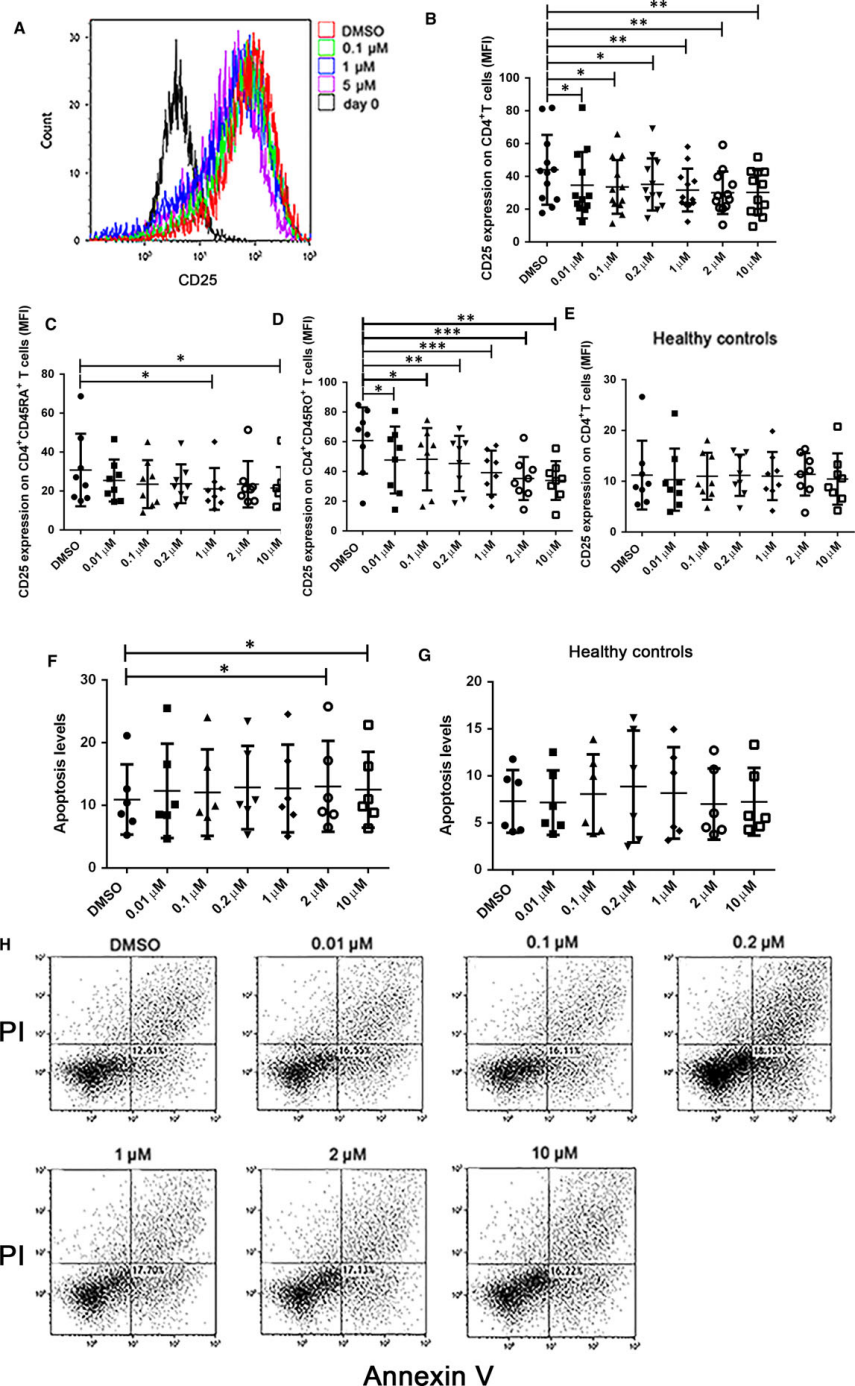
Indirubin dampened the activation of effector T cells in a dose‐dependent manner and induced apoptosis of peripheral blood mononuclear cells (PBMCs) at high doses. (A,B) Indirubin reduced the activation of CD4^+^ T cells in a dose‐dependent manner in the culturing of PBMCs from immune thrombocytopenia (ITP) patients. The CD25 expression on CD4^+ ^T cells was significantly reduced after indirubin treatment at the respective doses (0.01 µmol/L indirubin, 34.65 ± 20.23, *P* = 0.0407; 0.1 µmol/L indirubin, 33.57 ± 16.36, *P* = 0.0182; 0.2 µmol/L indirubin, 35.09 ± 15.85, *P* = 0.0413; 1 µmol/L indirubin, 31.66 ± 13.03, *P* = 0.0043; 2 µmol/L indirubin, 30.01 ± 12.98, *P* = 0.0039; 10 µmol/L indirubin, 30.20 ± 13.58, *P* = 0.0085; n = 12, respectively) compared with dimethyl sulfoxide (DMSO)‐treated groups (44.02 ± 21.3, n = 12). (C,D) Indirubin could reduce the CD25 expression on CD4^+^CD45RA^+ ^T cells at the doses of 1 and 10 µmol/L (1 µmol/L indirubin, 21.1 ± 10.74 vs 30.78 ± 18.63, *P* = 0.0269; 10 µmol/L indirubin, 21.59 ± 10.69 vs 30.78 ± 18.63, *P* = 0.0234, n = 8, respectively), and the expression of CD25 on CD4^+^CD25RO^+^ T cells could be reduced by indirubin in a dose‐dependent manner in PBMCs from ITP patients (DMSO 60.82 ± 22.30; 0.01 µmol/L indirubin 47.74 ± 22.49, *P* = 0.0292; 0.1 µmol/L indirubin 48.23 ± 20.97, *P* = 0.0279; 0.2 µmol/L indirubin 45.36 ± 18.57, *P* = 0.0052; 1 µmol/L indirubin 39.24 ± 14.77, *P* = 0.0006; 2 µmol/L indirubin 35.30 ± 14.53, *P* = 0.0006; 10 µmol/L indirubin 33.92 ± 13.06, *P* = 0.001, n = 8 respectively). (E) Indirubin did not affect the CD25 expression on CD4^+^ T cells in PBMCs from healthy controls. Differences between two groups were determined by paired Student's *t* test. (F) Indirubin induced the apoptosis of PBMCs from ITP patients at the dose of 2 and 10 µmol/L (DMSO 10.92 ± 5.59 vs 2 µmol/L indirubin 13.02 ± 7.23, *P* = 0.0493; 10 µmol/L indirubin 12.49 ± 6.04, *P* = 0.0295, n = 6). (G) No significant effect of apoptosis on PBMCs from healthy controls was observed. (H) The representative dot plots for the analysis of apoptosis in the culturing of PBMCs from ITP patients. The percentage of Annexin‐positive and PI‐negative cells on PBMCs represented the cell apoptosis rate. Differences between two groups were determined by Wilcoxon matched‐pairs signed rank test, *P < 0.05, **P < 0.01, ***P < 0.001

Whether in addition to the induction of Tregs expansion, indirubin could influence the function of effector T cells was unknown. In the culturing of PBMCs from ITP patients, we found the expression of CD25 in CD4^+^ T cells (Figure 2A,B), CD4^+^CD45RA^+^ T cells (Figure 2C) and CD4^+^CD45RO^+^ T cells (Figure 2D) were significantly reduced after treated with indirubin in a dose‐dependent manner, indicating that indirubin could decrease the activation of effector T cells in ITP. However, PBMCs from healthy controls did not show obvious changes in the activation of effector T cells after indirubin treatment (Figure 2E). We further tested the apoptosis level of PBMCs after treated with indirubin at different doses. We stained PBMCs with Annexin V and propidium iodide (PI) following co‐culturing with indirubin or DMSO for 72 hours. The percentage of apoptotic cells were considered as Annexin V‐positive and PI‐negative.^12^ Indirubin could only induce the apoptosis of PMBCs from ITP patients at higher doses of 2 and 10 µmol/L (Figure 2F,H), and the apoptosis level of PBMCs from healthy controls did not change much after indirubin treatment (Figure 2G). We then selected 1 µmol/L indirubin as the optimal dose as it could significantly induce the number and function of Tregs as well as dampen the activation of effector T cells without apparent induction of apoptosis.

### 3.3 **Indirubin normalized decreased PD1 and PTEN expression on CD4** ^+^ **T cells of ITP patients**

**Figure 3:**
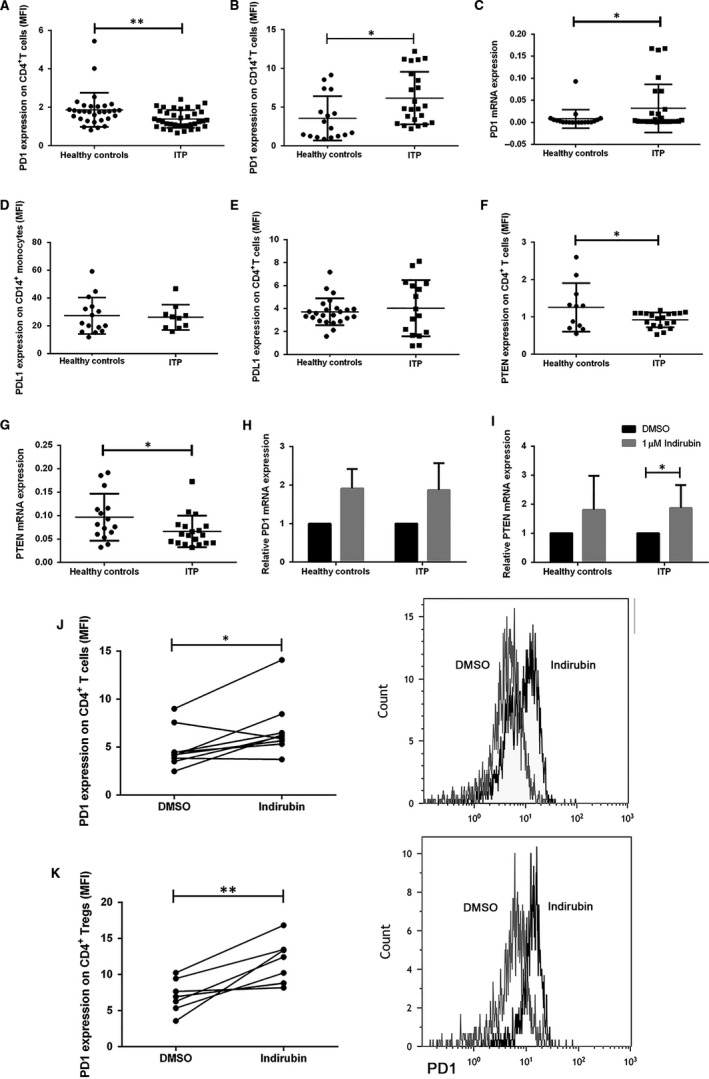
The reduced expression of programed cell‐death 1 (PD1) and phosphatase and tensin homolog (PTEN) in immune thrombocytopenia (ITP) patients was observed compared with healthy controls, and could be rectified by indirubin treatment. Peripheral blood mononuclear cells (PBMCs) were isolated from ITP patients and healthy controls to analyze the expression of PD1, PTEN using flow cytometry. (A) Decreased PD1 expression on CD4^+^ T cells (1.86 ± 0.89, n = 31 vs 1.39 ± 0.46, n = 37, *P* = 0.0063, Student's *t* test) and increased PD1 expression on CD14^+^ monocytes of ITP patients compared with healthy controls were observed (3.55 ± 2.86, n = 17 vs 6.16 ± 3.39, n = 23, *P* = 0.0144, Student's *t* test). (C) The mRNA expression level of PD1 on PBMCs from ITP patients was higher than that from healthy controls (0.0008 ± 0.01, n = 16 vs 0.0027 ± 0.03, n = 27, *P* = 0.0215, Mann–Whitney test). (E,F) The PDL1 expression on CD14^+^ monocytes and CD4^+^ T cells had no significant differences between ITP patients and healthy controls. (F,G) Phosphatase and tensin homolog expression on CD4^+^ T cells and PTEN mRNA expression level on PBMCs were significantly suppressed in ITP patients compared to healthy controls (1.26 ± 0.65, n = 11 vs 0.92 ± 0.20, n = 20, *P* = 0.0393; 0.096 ± 0.050, n = 15 vs 0.066 ± 0.034, n = 19, *P* = 0.0429, Student's *t* test). (H,I) Indirubin increased the PD1, PTEN mRNA expression on PBMCs from ITP patients (PTEN: 1.87 ± 0.62, *P* = 0.0269, n = 7, paired Student's *t* test). The PBMCs from healthy controls did not show significant changes in the PD1, PTEN mRNA expression level after indirubin treatment. (J,K) Indirubin significantly increased PD1 expression on CD4^+^ T cells (4.22 ± 2.35 vs 6.13 ± 1.98, n = 6, *P* = 0.0391, Wilcoxon matched‐pairs signed rank test) and Tregs (7.06 ± 5.31 vs 11.70 ± 9.21, n = 7, *P* = 0.0061, paired Student's *t* test).*P < 0.05, **P < 0.01

The PD1‐PDL1 pathway, a pivotal modulator of T‐cell homeostasis and Tregs development, is critical for limiting the immune response and thwarting the development of autoimmunity.^32^, ^41^, ^42^, ^43^ Not surprisingly, it has been reported that PD1 is found to be decreased on the surface of PBMCs of ITP patients.^39^ However, the expression of PD1 on CD4^+^ T cells in ITP patients has not been reported. We found reduced PD1 expression on CD4^+^ T cells and increased PD1 expression on CD14^+^ monocytes of ITP patients compared with healthy controls (Figure 3A,B). The mRNA expression level of PD1 on PBMCs from ITP patients was higher than that from healthy controls (Figure 3C). Furthermore, we also tested the PD1 ligand, PDL1 expression on CD14^+^ monocytes and CD4^+^ T cells, but no significant differences were observed (Figure 3D,E). Phosphatase and tensin homolog (PTEN), a signalling modulator downstream of PD1 activation, was significantly suppressed in ITP patients compared to healthy controls as evidenced by decreased PTEN expression on CD4^+ ^T cells and decreased PTEN mRNA expression on PBMCs (Figure 3F,G). To assess whether indirubin may ameliorate T‐cell dysfunction in ITP patients through normalization of PD1 and PTEN levels, we treated PBMCs from ITP patients or healthy controls with 1 µmol/L indirubin. Indirubin was found to significantly increase the PD1 expression on CD4^+^ T cells and Tregs in PBMCs from ITP patients (Figure 3J,K) with a concomitant increase in PD1, PTEN mRNA expression levels, though the increasing of PD1 mRNA expression was not significant (Figure 3H,I), thus indicating that indirubin's therapeutic action on Tregs and effector T cells might be through the induction of PD1 and PTEN expression and signalling in ITP. The PBMCs from healthy controls did not show significant changes in the PD1, PTEN mRNA expression level after indirubin treatment (Figure 3H,I), nor did the PD1 expression on CD4^+^ T cells (data not shown).

### 3.4 Indirubin ameliorated thrombocytopenia in an active murine model

**Figure 4:**
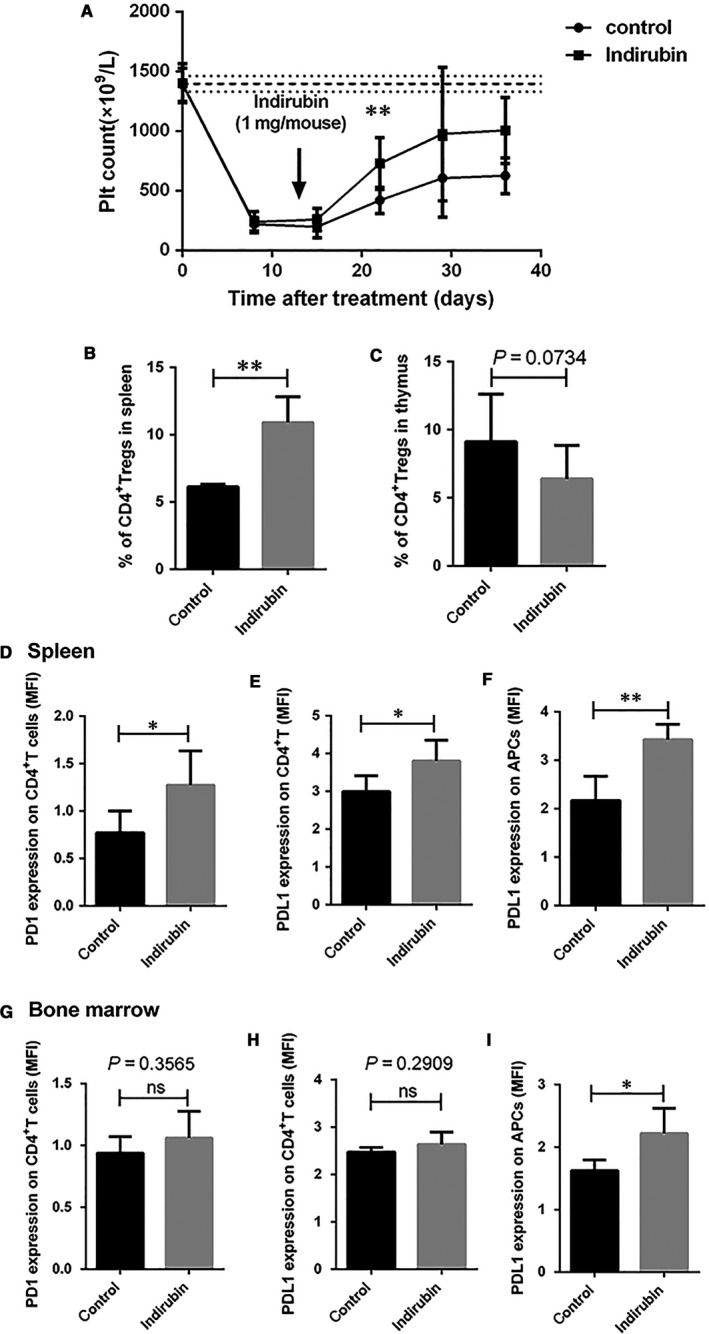
Indirubin ameliorated thrombocytopenia in an active immune thrombocytopenia (ITP) murine model with increased splenic Tregs and programmed cell‐death 1 (PD1) and PDL1 expression. (A) Immune thrombocytopenia was established in irradiated Severe combined immunodeficient mice with engraftment of 5 × 10^4^ splenocytes from CD61 KO mice immunized against wild type C57 mice platelets. Platelet counts were monitored every week for 5 wk. Treatment with indirubin (40 mg/kg) or control (coin oil) was administered on day 13. On day 21, a significantly higher platelet count was observed in the indirubin‐treated group compared to control group (728.33 × 10^9^/L ± 215.26 vs 403.33 × 10^9^/L ± 111.83; *P* = 0.0015). The horizontally dotted lines represent the normal platelet range. Significance among groups was determined by Student's *t* test, n = 6 and 7 for indirubin and control group respectively. Spleens, thymuses and bone marrow were harvested from ITP mice at 35 d post splenocytes transfer (23 d after indirubin treatment). (B,C) indirubin‐treated ITP mice had a higher percentage of splenic Tregs (6.14 ± 0.19 vs 10.92 ± 1.90, *P* = 0.005) and lower percentage of thymic Tregs (9.12 ± 3.56 vs 6.39 ± 2.46, *P* = 0.129) compared with the control ITP mice. (D‐I) The expression of PD1 (0.77 ± 0.23 vs 1.27 ± 0.36, *P* = 0.047), and PDL1 (2.99 ± 0.42 vs 3.8 ± 0.55, *P* = 0.045) on CD4^+^ T cells and the expression of PDL1 on antigen presenting cells (APCs) (2.17 ± 0.50 vs 3.42 ± 0.32, *P* = 0.002) were upregulated in the spleen, as well as PDL1 on APCs from bone marrow (1.63 ± 0.17 vs 2.22 ± 0.40, *P* = 0.027), whereas no significant changes of PD1, PDL1 expression on CD4^+^ T cells from bone marrow. Differences between two groups were determined by Student's *t* test, n = 4 for control group, and n = 5 for indirubin group. *P < 0.05, **P < 0.01

It has been reported previously that indirubin was effective therapeutically in an ITP murine model.^31^ However, the animal model used in the previous study could not explain the pathogenesis of ITP sufficiently. To assess whether our in vitro observations were reproducible in vivo, we adopted an active ITP murine model. This is currently the optimal model in mimicking human chronic ITP as it encompasses both antibody‐mediated and cell‐mediated platelets and megakaryocytes destruction.^44^ Following the radiation and transfer of anti‐CD61 immune‐sensitized splenocytes into SCID mice, platelet counts dropped to nadir on day 7. Indirubin (40 mg/kg) or control treatment (corn coil) was administered via intraperitoneal injections on day 13. On day 21, significantly higher platelet counts were observed in the indirubin‐treated group compared to control group (Figure 4A). To further investigate the molecular mechanisms of therapeutic efficacy of indirubin in vivo, we quantified the percentage of CD4^+^CD25^+^Foxp3^+ ^Tregs from the spleen and thymus as well as the expression of PD1, PDL1 in the ITP mice. Compared with the control group, indirubin treatment increased splenic Tregs 35 days after splenic transfer (23 days after indirubin treatment) and reduced the thymic Tregs (Figure 4B,C). The PD1 expression on CD4^+^ T cells was upregulated in the spleens of indirubin‐treated group (Figure 4D). Moreover, PDL1 was increased on splenic CD4^+^ T cells and CD80^+^CD86^+^ APCs after indirubin therapy (Figure 4E,F).

### 3.5 Indirubin regulated the homeostasis of CD4^+^ T cells via a PTEN/AKT/mTOR signalling pathway

**Figure 5:**
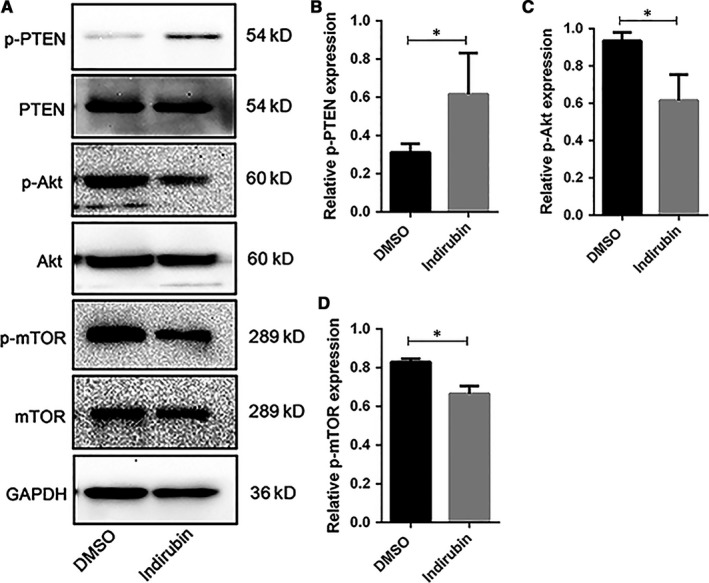
Indirubin activated phosphatase and tensin homolog (PTEN) and inhibited the AKT/mTOR signalling pathway in CD4^+^ T cells. (A) CD4^+^ T cells isolated from immune thrombocytopenia peripheral blood mononuclear cells were treated with indirubin or dimethyl sulfoxide (DMSO) control. Western blotting was performed on cell lysates. (B‐D) Graphs present densitometry data of relative phosphorylated (p‐) protein to total protein after indirubin or DMSO treatment (p‐PTEN 0.31 ± 0.052 vs 0.68 ± 0.14, *P* = 0.025; p‐AKT 0.94 ± 0.033 vs 0.66 ± 0.082, *P* = 0.0452; p‐mTOR 0.81 ± 0.054 vs 0.63 ± 0.10, *P* = 0.0497. Paired Student's *t* test, n = 3, *P < 0.05

The downstream signalling events of PD1 include augmenting PTEN expression which consequently leads to attenuation of the phosphatidylinositol 3‐kinase (PI3K)/AKT/mTOR pathway.^45^, ^46^, ^47^, ^48^ This process modulates a plethora of cellular processes including energy metabolism and plays a pivotal role in the regulation of autoimmunity, inflammation, and cancer.^48^, ^49^, ^50^, ^51^ Western blot analysis of cell lysates from CD4^+^ T cells cultured with 1 µmol/L indirubin revealed increased PTEN phosphorylation and decreased AKT and mTOR phosphorylation (Figure 5). These results suggested that indirubin could restore the homeostasis of CD4^+^ T cells in a PTEN/AKT/mTOR signalling pathway which is important in the maintenance of CD4^+^ T‐cell homeostasis in ITP.

## 4 DISCUSSION

*I tinctoria *L. is an herb used in ancient China for the treatment of thrombocytopenia. Its effect is mild and persistent, and therefore is usually used as an assistant therapy for the treatment of ITP nowadays. Indirubin is an active ingredient of *I tinctoria *L., and is well known for its anti‐cancer and anti‐inflammatory effects.^25^ In addition, indirubin has also been reported to be an effective treatment in autoimmune diseases.^52^ ITP is a multifaceted autoimmune disease, and its complex pathogenic mechanisms still remain to be fully elucidated. One of the major contributing factors to the immuno‐pathogenesis of ITP is the disturbance of the T‐cell homeostasis including the peripheral deficiency of Tregs and over‐activation of effector T cells.^5^, ^6^, ^7^, ^8^, ^14^, ^15^, ^16^ In animal experiments, indirubin was found to increase the percentage and number of Tregs in murine models of ITP and ulcerative colitis. 27, 31

In our present study, we showed that indirubin enhanced Tregs development and improved Tregs function of ITP patients in vitro. Beyond its effects on Tregs, indirubin also exerted a regulatory role on CD4^+ ^effector T cells as dampening the activation of effector T cells. Furthermore, we found lower PD1 and PTEN expression in CD4^+^ T cells from ITP patients compared to healthy controls, which could be rectified after indirubin treatment. The PD1‐PDL1 pathway regulates peripheral T‐cell tolerance in several ways. On the one hand, this pathway impairs the initial phase of activation, expansion, differentiation, survival, and functions of self‐reactive T cells. On the other hand, this pathway facilitates the development, maintenance, and function of induced Tregs.^33^, ^53^, ^54^, ^55^ Signalling through the PD1‐PDL1 pathway also regulates the dynamic interplay between Tregs and effector T cells.^56^ The combination of PD1 and PDL1 limits T‐cell stimulation and promotes the differentiation and stabilization of Tregs by augmenting the expression of PTEN, a cellular phosphatase which inhibits the PI3K/AKT/mTOR pathway.^45^, ^46^, ^47^, ^48^ The reduced expression of PD1 and PTEN on CD4^+^ T cells of ITP patients may contribute to the disturbance of CD4^+^ T‐cell homeostasis in ITP. In our in vitro study, indirubin was found to increase the PD1 expression on CD4^+^ T cells of ITP patients, and induce the phosphorylation of PTEN, with the downstream dephosphorylation of p‐AKT and p‐mTOR.

We established the active murine ITP model, and significant amelioration of thrombocytopenia was found 7 days after indirubin administration, implicating a therapeutic role of indirubin in ITP. It has been reported in murine models of ITP that thrombocytopenia was correlated with a significant splenic Tregs deficiency and a concomitant thymic retention of Tregs, which was reversed upon amelioration of ITP.^22^ Similarly, in our murine ITP model, we found successful indirubin treatment correlated with an increase of splenic Tregs percentage and a concurrent reduction in the thymic Tregs. Furthermore, in accordance with our in vitro study, our in vivo study revealed the upregulated expression of PD1 on CD4^+^ T cells in the spleens from ITP mice treated with indirubin. Moreover, the expression of PDL1 was also increased on the CD4^+^ T cells and APCs after indirubin treatment.

To the best of our knowledge, this is the first time to demonstrate that indirubin may exert its effects through the PD1‐PDL1 axis which has a significant role in reinstating T‐cell homeostasis and re‐establishing peripheral tolerance. In our future study, we plan to use the PD1^‐/‐^ mice to establish the ITP murine model and further elucidate the correlation between ITP and PD1 signalling pathway, and the mechanisms of indirubin in a deeper facet. We also plan to register a randomized controlled clinical trial to investigate the therapeutic effect of indirubin on ITP patients clinically.

In summary, indirubin modulated the T‐cell homeostasis by enhancing the development and function of Tregs and reducing the activation of effector T cells via a PD1/PTEN/AKT signalling pathway. This novel mechanism of indirubin underlies the potential therapeutic strategy for ITP.

## CONFLICT OF INTEREST

The authors confirm that there are no conflicts of interest.
